# HopBase: a unified resource for *Humulus* genomics

**DOI:** 10.1093/database/bax009

**Published:** 2017-04-06

**Authors:** Steven T. Hill, Ramcharan Sudarsanam, John Henning, David Hendrix

**Affiliations:** 1 Electrical Engineering and Computer Science, Oregon State University; 2 USDA-ARS-Forage Seed & Cereal Research, Corvallis, OR 97331, USA; 3 Biochemistry and Biophysics, Oregon State University, OR, USA

## Abstract

Hop (*Humulus lupulus L. var lupulus*) is a dioecious plant of worldwide significance, used primarily for bittering and flavoring in brewing beer. Studies on the medicinal properties of several unique compounds produced by hop have led to additional interest from pharmacy and healthcare industries as well as livestock production as a natural antibiotic. Genomic research in hop has resulted a published draft genome and transcriptome assemblies. As research into the genomics of hop has gained interest, there is a critical need for centralized online genomic resources. To support the growing research community, we report the development of an online resource "HopBase.org." In addition to providing a gene annotation to the existing Shinsuwase draft genome, HopBase makes available genome assemblies and annotations for both the cultivar “Teamaker” and male hop accession number USDA 21422M. These genome assemblies, gene annotations, along with other common data, coupled with a genome browser and BLAST database enable the hop community to enter the genomic age. The HopBase genomic resource is accessible at http://hopbase.org and http://hopbase.cgrb.oregonstate.edu.

## Introduction

Hop is a plant of great cultural significance, used as a medicinal herb for thousands of years and as a key ingredient in brewing beer for flavoring and as a preservative (1–3). Hop is a large, climbing, dioecious plant in the Rosid class. The *Humulus* genus contains three species, *Humulus japonicus, Humulus lupulus* and *Humulus yunnanensis*, two of which, *H. japonicus* and *H. lupulus*, are known to produce compounds with beneficial pharmaceutical properties (4). Little is known about *H. yunnanensis* and it may be extinct, even though there has been effort to find a living plant (5). *Humulus* also has three typical sex chromosome configurations: *H. lupulus* (2n  =  18  +  XY), *H. lupulus var. cordifolius* (2n  =  16  +  X1X2 Y1Y2) and *H. japonicus* (2n  =  14  +  XY1Y2) (6). The simplicity of *H. lupulus var. lupulus* makes possibly the more tractable of these configurations for genome assembly. These configurations provide an interesting platform for studying sex chromosome evolution in plants and several research projects have been focused around this already (7, 8).

Cytogenetic research and genome assembly analysis suggest that the hop genome is approximately 2.8 Gb and highly repetitive (9, 10). Large amounts of repetitive DNA cause difficulties in short-read genome assembly due to the inability to assemble through repetitive regions. As a result, the repeat regions are larger than current mate-pair technology and require expensive long-read sequencing methods to assemble. Efforts using short-read sequencing techniques have been extensive and exhaustive and the resulting assemblies, while incomplete, are now available (10).

Currently, there exists published work on a high marker density genetic map (11), several RNA sequencing datasets (10, 12), a draft genome assembly, a plethora of research surrounding the essential oils, and many other secondary resources (13–15). Furthermore, we have deep-sequenced, assembled, and annotated another female hop variety, Teamaker (16), The assembly was used to guide the assembly of the first male hop genome (USDA 21422M) coupled with the identification of male-specific DNA and pseudo-autosomal regions of the sex chromosomes (7). None of these resources include a public annotation, and no attempt has been made to consolidate this information into a single resource. Standardizing data and providing a unified access are a core challenge in genome annotation and bioinformatics (17–19). The consolidation of information allows for a much cleaner and easier flow of information among hop researchers. The objective of our work was to assemble both a male and female hop genome and to couple the information from these assemblies along with all other online hop genome information into a single resource available to hops researchers and breeders alike.

## Materials and methods

### Teamaker genome assembly

The Teamaker genome assembly used libraries selected in accord to the ALLPATHS-LG recipe (20). Reads were adapter trimmed and filtered for a mean quality of at least 30 using the program Skewer (21). Duplicated reads were removed using a custom C ++ program (https://github.com/hillst/dedup_paired_reads). The dedup process simply collapses read pairs that are completely identical for both mates. This resulted in an estimated coverage of 109× (Table 1). Assembly was performed using the ALLPATHS-LG assembly with ploidy of two and using a minimum contig size of 500. Lower minimum contig settings resulted in infeasible computation time and memory usage. The assembly took approximately 1 month to complete on a 64 CPU machine with 512GB of memory using the remaining 3 billion reads (Table 1). The resulting assembly was gap filled using GapCloser 1.0 (22). Hereafter, we will refer to this assembly as the preliminary genome assembly.

**Table 1.** Sequencing libraries used for the Teamaker genome assembly

Mate pair insert size (bp)	Number of sequenced reads	Number of single-copy + QC Reads	Portion removed from dedup + QC	Estimated coverage
9000	796 503 434	164 452 668	0.794	6.091
6000	363 664 930	96 117 630	0.736	3.560
5000	830 281 020	611 993 950	0.263	22.666
3000	618 181 114	379 821 668	0.386	14.067
**Mate pair Total**	2 608 630 498	1 252 385 916	0.520	46.385
**Fragment library insert size (bp)**				
143	1 655 421 082	708 994 796	0.572	26.259
173	1 176 857 672	606 418 512	0.485	22.460
250	419 621 690	388 494 910	0.074	14.389
**Fragment total**	3 251 900 444	1 703 908 218	0.476	63.108

### Transcript guided assembly

**Figure 1.**
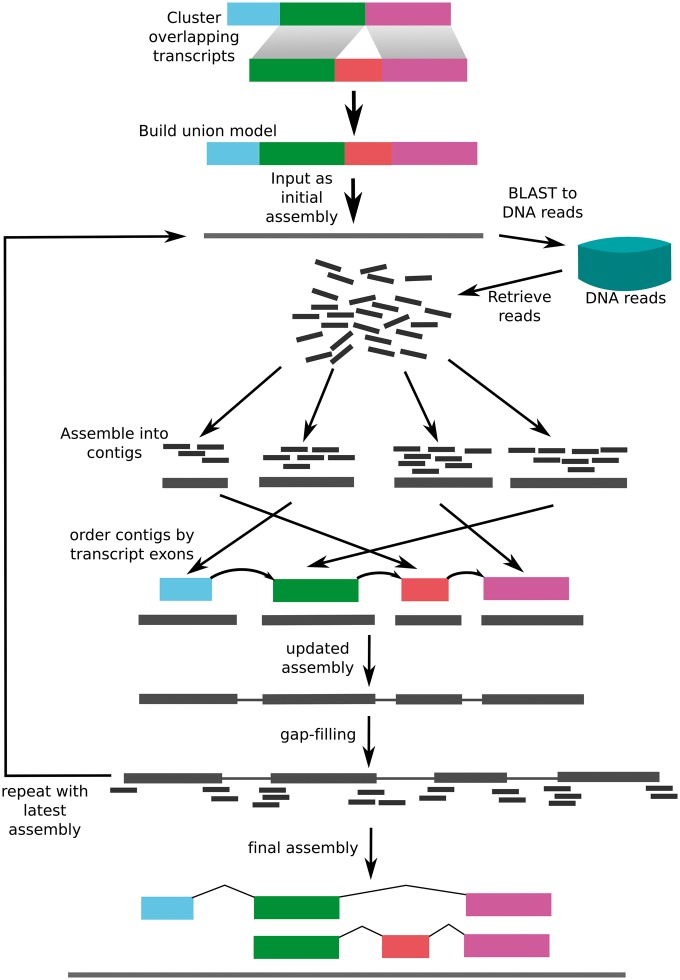
The transcriptome-guided Assembly (TGA) pipeline. Transcripts are combined to form a union model consisting of all exons present for each isoform. The resulting sequence is used as the initial “assembly.” The assembly is aligned to the DNA reads using BLAST, and all aligning reads are retrieved. The Reads are assembled using Velvet, and ordered according to the order of the corresponding exons in the transcript models. After gap filling, this process is repeated until subsequent applications do not change the assembly.

Transcript guided assembly (TGA) is an approach to improving genome assemblies that exploits the fact that transcripts contain order information about the genome, similar to a mate-pair read (Figure 1). To make use of this information, the assembled mRNA sequences are used to retrieve DNA reads corresponding to the genomic sequence overlapping their corresponding genes. This results in an assembly of the genomic sequence overlapping and adjacent to the genes, which contains partial or complete promoters, introns and other flanking sequence. These regions that would otherwise be broken by repeats in introns are also properly ordered in the resulting assembly. Our approach is similar to a recently published approach (23) but with the addition of contig ordering and gap closing.

We used SOAPdenovo-trans (http://soap.genomics.org.cn/SOAPdenovo-Trans.html) to perform a *de novo* transcriptome assembly to perform the first step of transcriptome-guided assembly (24). RNA-seq reads were acquired from the Shinsuwase publication (10). All RNA-seq libraries corresponding to the cultivar Shinsuwase were downloaded from the DNA Databank of Japan ID: DRA002630. The resulting assembly was 1 102  071 scaffolds with an N50 of 431, indicating many small scaffolds. The contigs were filtered to a minimum length of 1000 bp in order to remove most of the scaffolds consisting of partial gene fragments. The remaining contigs were then filtered for contaminants using BLAST against the NR database (25). Statistically significant hits that were not to plant sequences were removed. This resulted in 43  926 scaffolds with an N50 of 2765. We then aligned these transcript sequences to the preliminary genome assembly. Transcripts that did not align were used for TGA. Whole genome reads were aligned to these transcripts using BLASTN. These reads were then assembled using Velvet with a K of 51 and otherwise default parameters (26). The resulting contigs were aligned back to the original transcript using Exonerate (27). The result was considered to be the "order" of the assembled contigs. Contigs were ordered and scaffolded together with Ns separating each contig. The gaps were then filled using GapCloser 1.0. This process was repeated five times for each transcript. The result was a final assembly of 1 766  890  029 bp with an NG50 of 41 006 bp.

The potential limitation of the TGA approach is that there can be misassembles of transcripts that will ultimately lead to errors in the genome assembly (28). We addressed this by stringently filtering out transcript models by length and filtering out any resulting contigs that do not significantly align to plant genes in NR. The fact that performing TGA led to an improvement of public hop ESTs compared to the Shinsuwase assembly provides some validation of the method.

### Repeat library construction

Novel repeats were constructed according to a process whereby k-mers that have a high copy number selected to assemble a repeat library (29). Next, we identified high-copy k-mers in the 173-bp library using Jellyfish (http://www.cbcb.umd.edu/software/jellyfish/) and *k*  =  31 (30). The k-mers that had >120 copies, which is 6 times the expected coverage, were labeled as repetitive. These k-mers were then assembled using velvet to give an initial set of repeat sequences. Sequences of < 64 bp were removed. The remaining sequences were aligned using BLAST against the MIPS repeat element database (mips-REdat) and NR for identification (31). Contigs with alignments to chloroplast, mitochondria and rRNA were placed into their own categories. Sequences with a functional annotation to plants that were not repeats were also removed from the library and marked for future analysis. The final set of repeats had an N50 of 212 and contained 9615 repeats. These repeats were then annotated using pre-trained models of TE-class (32). TE-class uses hierarchical classification; it classified 98% of repeats, 85.8% of the retrotransposon class and 14.2% of the DNA transposon class (Table 2). This is in accordance with other angiosperms. This library was combined with mips-REdat to create the final repeat library for use in masking.

**Table 2.** Distribution of repeats in Teamaker assembly by length

	100–200bp	201–300bp	301bp+	Total
LTR	2094	780	353	3227
Unclear	155	51	52	258
DNA Transposon	1024	240	60	1324
Retro	1533	545	212	2290
LINE	303	601	618	1522
SINE	621	62	3	686
nonLTR	235	67	6	308

### Shinsuwase and Teamaker assembly annotation

The genome annotation was performed in a multi-step fashion. First, the genome was masked using RepeatMasker along with the previously described repeat database, and the remaining unmasked genome was annotated further (33). The RNA-seq reads described previously were aligned to the genome assembly using HISAT version 0.1.6 and a transcriptome assembly was constructed using StringTie version 1.2.0 (34, 35). This resulted in over 1 000  000 transcripts that are much higher than expected, likely due to the unusually high volume of RNA-seq (Table 3). Most of the genes were single exon with low read coverage and thus likely to be spurious. Genes were filtered using outlier detection via one-class SVM trained using scikit-learn (36). Outliers were then called genes and used as the first set of genes. MAKER-P (http://www.yandell-lab.org/software/maker-p.html), a pipeline for the automatic annotation of plant genomes, was then run on the masked genome with the StringTie transcripts used as external information (37). Augustus and SNAP were used as gene finders with the provided *Arabidopsis* models (38, 39). Finally, the peptide sequences of the remaining genes were extracted and aligned to the TAIR10 Arabidopsis mitochondria and chloroplast protein sequences using BLASTP (40, 41). We required an E-value less than or equal to 1E-4 for subsequent analysis. We further separated all genes that contained the keywords “gag,” “pol,” “Retrotransposon” and “Retroelement” from the core annotation.

**Table 3.** Gene annotation for Shinsuwase and Teamaker assemblies

	Shinsuwase	Teamaker
StringTie Transcripts	1 120 693	1 137 597
StringTie w/SVM Transcripts	97 288	77 118
MAKER genes	46 735	39 831
MAKER after pseudogene removal	39 672	28 434
MAKER after repeat removal	35 482	24 919
Genes with unknown protein homology	13 281	8758
Genes with protein homology	22 201	16 161
Total remaining genes	35 482	24 919

The remaining genes were then scanned for functional annotations using BLASTP against a database of known hop genes, TAIR 10, and against Uniprot (41, 42). The annotation with the lowest E-value was selected. This gave a set of 22  201 and 16 161 annotated genes in the Shinsuwase and Teamaker annotations, respectively (Table 3). The difference in total genes and annotated genes can be characterized by the difference in assembly methods. ALLPATHS-LG is known to be a conservative assembler, possibly excluding highly heterozygous genes or broken genes. Similarly, an aggressive assembler may include these genes as two separate scaffolds. In any case, it is clear much work needs to be done before the hop draft genome can be called complete.

### 21422M annotation

The genome assembly of 21422M was the same used in our previous work identifying the pseudo-autosomal region (7). The genome was annotated in a simpler fashion, as the identification of complete genes is not as confident in a genome with low sequencing coverage. The previously mentioned RNA-seq reads were quality filtered with a mean quality of 30 and adapters were removed using Skewer (12, 21). Reads were then aligned using HISAT to the 21422m assembly (7, 34). Transcript models were assembled using StringTie.

### System implementation

**Figure 2.**
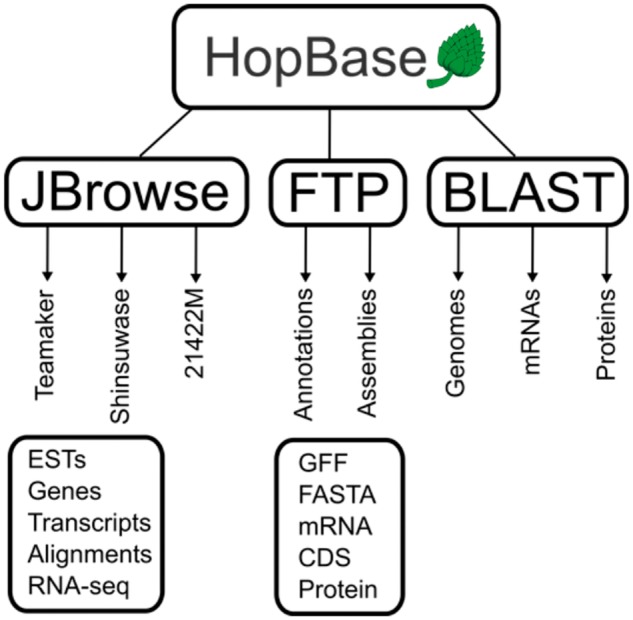
A schematic representation of HopBase. HopBase consists of three genome assemblies including Teamaker, Shinsuwase and a male accession number 21422M. There is a JBrowse genome browser for each assembly, as well as FTP site for downloading sequences and annotation files for each assembly. We also provide a BLAST interface for aligning sequences to mRNA, protein and genome collections.

HopBase is a web-based resource for these assemblies and annotations. The server itself is a 32 AMD-x64 CPU machine with 32 Gigabytes of RAM and a 10 Gigabit connection to the Oregon State University ISP. The HopBase software stack consists of, Linux CentOS 6.6 final, Apache2, PHP5, Symfony2, Bootstrap3 and AngularJS. The use of modern front-end libraries, specifically AngularJS 1.0 and Bootstrap provides a modern look-and-feel for HopBase while Symfony provides maintainable backend architecture using a mature Model-View-Controller (MVC) framework. The three assemblies available are USDA 21422M, Shinsuwase and Teamaker (7, 10, 16). Each assembly includes an annotation using the RNA sequence data provided by Natusme *et al.* A schematic of the structure of the web interface of HopBase is presented in Figure 2.

### BLAST

The BLAST web tool is implemented using SequenceServer (43). SequenceServer is a standalone tool for interfacing with the command line NCBI BLAST (http://www.sequenceserver.com/). The databases included on the website correspond to each genome assembly, coding sequences, predicted protein sequences, and other specialty databases. In particular, the male specific region is a standalone BLAST database. Access to an easy-to-use BLAST interface specific to hop will greatly help the hop research community.

### Resources

The resources page hosts raw data for bulk download: files for genome assemblies, various annotation formats and other processed resources (VCF, BAM, gene expression). It also includes the standardized ID format for submission from users. Downloading and accessing the raw files for bioinformatics can be a challenge, especially when there are multiple resources present as well as locations for these resources. A central location containing each of the aforementioned files provides scientists an easy starting point for working on *Humulus* genomics. While there are advantages of more general genetic resource databases, such as the ability to integrate data across many plant systems (44, 45), HopBase provides genomics resources specifically focused on one plant system, thereby providing content aimed at researchers of hop.

### JBrowse

**Figure 3.**
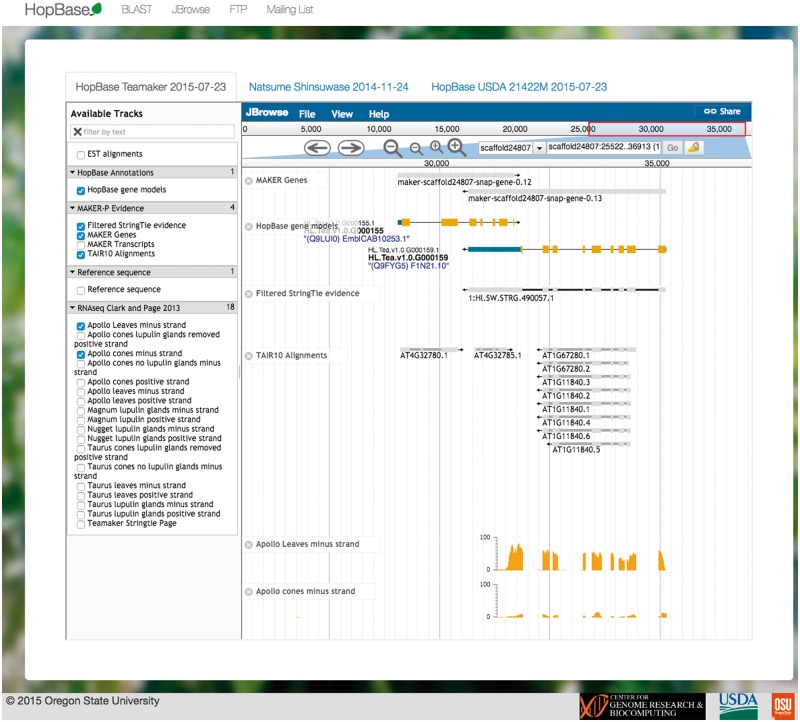
HopBase provides a JBrowse genome browser consisting of multiple tracks such as gene models, ESTs, alignments from TAIR, and RNA-seq data.

The JBrowse server is hosted on the same machine at http://jbrowse-hopbase.cgrb.oregonstate.edu (46). Each genome assembly is provided as a separate tab within the front-end framework. This allows for quickly switching between contexts and allowing for the data to be loaded in the background. Each JBrowse interface includes the final annotations, the StringTie assembly, repeat annotations, gene expression for each available tissue type, as well as predicted motifs for known plant transcription factor binding sites. In addition, JBrowse includes RNA-seq experimental expression data for genes and known transcripts across several different hop varieties. An example of a genomic locus displayed with the JBrowse interface is shown in Figure 3.

### Mailing list

The HopBase mailing list provides for rapid information regarding updates when pushed to production. If a new annotation is produced, or a new draft of the genome is available, it is easy to notify users of this information. This provides a convenient alternative to frequently checking the website for updates.

## Results

Genome assemblies for both a male and female hop accession were developed and fully annotated to the degree possible given the repetitive nature of the hop genome and the difficulties associated with said assembly. Overall sequencing depth for Teamaker was 209X prior to read processing (Table 1). Fragment libraries (101 bp) had insert sizes of 143 bp, 173 bp and 250 bp. This resulted in 63.1X coverage after removal of duplicates and quality control. In addition, mate-pair, paired-end reads (101 bp) with insert sizes ranging from 3000 to 9000 bp were sequenced for an additional coverage of 46×. Sequencing libraries with insert sizes outside normal library preparation of approximately 250 bp insert size proved difficult to develop and losses due to quality control reflected this. Ultimately, the total coverage for sequencing Teamaker after removal of duplicated reads and quality control was approximately 109×.

The Teamaker genome assembly has similar assembly statistics with that published for the Shinsuwase genome with each having their respective strengths and weaknesses (Table 4) (10). The Teamaker assembly has slightly higher alignment to transcriptome assembly while alignments to Public EST data are slightly higher with the Shinsuwase genome. The Shinsuwase genome also has a slightly higher alignment to CEGMA core genes than Teamaker. It is likely that the higher alignment of Teamaker with public transcriptome data is due to the use of transcriptome-guided genome assembly as an aid to assembling the genome. Finally, the Teamaker genome (with Ns) is closer to actual size than that observed for Shinsuwase. Gene annotation was more successful using the Shinsuwase genome assembly with the exception of StringTie Transcripts (Table 3).

**Table 4.** Comparison of Shinsuwase assembly and Teamaker assembly

	Shinsuwase v1 (10)	Hopbase Teamaker v1(current)
Transcriptome Assembly alignments	70%	76%
Public ESTs alignments	94%	96%
CEGMA genes	89%	85%
NG50 (without Ns)	5050	9231
NG50 (with Ns)	N/A	41 006
Assembly size (with Ns)	2 049 209 000	2 770 850 934
Assembly size (without Ns)	1,775,776,000	1,766,890,029

One feature common to both assemblies is the presence of large numbers of DNA repeats (Table 2). These repeats varied in size from 100 bp to > 300 bp. The vast majority of repeats consisted of long terminal repeats (LTR) and retro-transposons. The next group, long-interspersed-nuclear-elements (LINEs) made up the majority of repeat sequences that were >300 bp in length. Finally, large numbers of DNA transposons were observed with most ranging in size from 100 to 200 bp. It is likely that a significant portion of the unassembled regions of both genomes consists of repetitive elements. It is observed that regions on the boundary of scaffolds had a much higher copy number than portions in the center of scaffolds, further indicating that the assembled regions are bordered by repeat regions.

In-house development of genetic linkage maps demonstrated Teamaker as superior for use in identifying SNP markers that could be mapped to linkage groups. Genetic maps for a population segregating for short stature hops were made using SNPs identified using reference-guided TASSEL v 3.0 pipeline (47). In the case of SNP markers identified using the Shinsuwase genome, only 677 markers mapped to 10 different linkage groups (11). Use of Teamaker genome assembly for SNP identification under the same default conditions as used for Shinsuwase resulted in a genetic map with 1531 markers mapped to 10 different linkage groups. The same phenomenon was observed in the development of a genetic map for a population segregating for downy mildew resistance (data not shown). These observations are reported not as a means of accessing assembly quality but as a suggestion for use in identifying markers for linkage or association mapping studies.

### Relatedness of cultivars

SNPs were called from 15× of whole genome sequencing reads for the cultivars Teamaker, Shinsuwase, USDA 21422M and Cordifolius. SNPs were all called using GATK and the corresponding best practices pipeline. Co-ancestry was computed using the relatedness phi, implemented in vcftools (http://vcftools.sourceforge.net/); large negative values indicate individuals from different populations, whereas positive values within a population are an approximation of the kinship coefficient (48, 49). From these statistics, the fact that Shinsuwase, Teamaker and USDA 21422M are from the same population is a given and is widely accepted among hop breeders (Table 5). In addition, Teamaker and USDA 21422M are clearly from a different population than Cordifolius, which again is accepted among hop breeders. However, Shinsuwase and Cordifolius have a relatedness score of nearly 0, which indicates unrelated individuals within a population. While the sample number is low, the genotype data suggest a relationship between Cordifolius and Shinsuwase that is not shared among other cultivated hops.

**Table 5.** Estimates of co-ancestry as calculated by use of phi (Manichaikul *et al.*, 2010)

INDV1	INDV2	N_AaAa	N_AAaa	N1_Aa	N2_Aa	PHI
USDA21422M	USDA21422M	316 418	0	316 418	316 418	0.5
USDA21422M	Cordifolius	26 110	79 853	316 418	53 545	−0.361106
USDA21422M	Shinsuwase	237 926	1821	316 418	389 779	0.331754
USDA21422M	Teamaker	245 479	18 564	316 418	324 193	0.325238
Cordifolius	USDA21422M	26 110	79 853	53 545	316 418	−0.361106
Cordifolius	Cordifolius	53 545	0	53 545	53 545	0.5
Cordifolius	Shinsuwase	20 020	20 108	53 545	389 779	−0.0455558
Cordifolius	Teamaker	23 928	55 859	53 545	324 193	−0.23241
Shinsuwase	USDA21422M	237 926	1821	389 779	316 418	0.331754
Shinsuwase	Cordifolious	20 020	20 108	389 779	53 545	−0.0455558
Shinsuwase	Shinsuwase	389 779	0	389 779	389 779	0.5
Shinsuwase	Teamaker	247 013	963	389 779	324 193	0.343273
Teamaker	USDA21422M	245 479	18 564	324 193	316 418	0.325238
Teamaker	Cordifolious	23 928	55 859	324 193	53 545	−0.23241
Teamaker	Shinsuwase	247 013	963	324 193	389 779	0.343273
Teamaker	Teamaker	324 193	0	324 193	324 193	0.5

## Discussion

It is likely that much of the missing portions of both genomes are repetitive elements. It is observed that regions on the boundary of scaffolds had a much higher copy number than portions in the center of scaffolds. The creation and unification of hop genomic resources provide a centralized location for hosting future genomic assemblies and annotations. Furthermore, it is possible to compare and contrast the different draft genomes and even ultimately repair and clean them when a complete genome assembly is available.

There are differences between the two assemblies. Our analysis resulted in the Shinsuwase assembly to be annotated with a higher number of genes than the Teamaker genome. In addition, the RNA-seq dataset had a higher percentage of alignments to the Shinsuwase genome. This latter result is expected given that RNA-seq data used for annotation came from Shinsuwase. Perhaps the simplest explanation for differences between genome assemblies is the format of the assemblies themselves. The Shinsuwase assembly was published with all gaps reduced to a single “N,” which could cause spurious gene isoforms called from the different gene finding software.

Another explanation for the discrepancy between the two genome assemblies is lineage of the two different varieties used for sequencing. Shinsuwase was an offspring of open pollenated “Saazer” variety grown in Japan. It is possible that the male parent for this cross has in its lineage *H. lupulus var cordifolius.* Teamaker arose from a cross between two parents possessing only *H. lupulus* var *lupulus* in their respective lineages. Genetic distances computed from SNPs within the deep sequencing of Teamaker, USDA 21422M, Shinsuwase and *H. lupulus var cordifolius* suggest that this is the case. Shinsuwase is by far the cultivar most closely related to the wild Japanese hop (Supplementary data).

The discrepancy between the two assemblies could also be due to the use of different assembly methods. The Shinsuwase assembly was performed using Celera assembly cell and the SSPACE scaffolder. In contrast, the Teamaker assembly was performed using ALLPATHS-LG. It is well known that ALLPATHS-LG is a more conservative assembler and scaffolder than the combination of CLC assembly cell and SSPACE. Groups who used CLC or SSPACE (no group used both) and participated in Assemblathon 2 performed worse in quality metrics on average than groups that used ALLPATHS-LG (50). In contrast, these groups performed as well or better than ALLPATHS-LG groups when measured on continuity (N50). In other words, ALLPATHS-LG will produce higher quality, yet smaller and shorter genome assemblies (conservative), while alternative methods will result in lower quality yet longer and larger assemblies (greedy).

The differences between assembly methods also provide potential cause for the discrepancy in the number of genes. Perhaps the simplest explanation is the format of the genome assemblies themselves. If an assembler program is more conservative about separating different haplotypes—especially large insertions or deletions—it would be less likely to duplicate genes which appear only once within the genome. On the contrary, a less conservative assembler program would be more likely to incorrectly separate single genes into multiple genes in the presence of large insertions or deletions. Furthermore, a less conservative or “greedy” approach to assembly may identify genes that are only partially sequenced whereas a conservative approach might not report the presence of such a partially sequenced gene.

While both approaches have their respective advantages, it is more useful and constructive to consider the cases in which each is useful. The greedy approach is more useful when researchers require a low false-negative rate at identifying regions of the hop genome. An example could be gene expression quantification with RNA-seq. The more conservative method is when you need high resolution of the hop genome and a low false positive rate of assembled regions. An example would be researchers who are interested in the genotypes of different hop cultivars.

The final difference between assembly methods is related to the transcriptome guided genome assembly of missing genes from the Teamaker assembly. Since the target genes were directly taken from the transcriptome, which was filtered for contaminants, it is expected that the Teamaker assembly would contain a higher number of EST and transcriptome alignments, as demonstrated in Table 4.

## Funding

Funding was provided by USDA-ARS CRIS #5358-21000-040-00D and Oregon State University.
